# Applying ‘drought’ to potted plants by maintaining suboptimal soil moisture improves plant water relations

**DOI:** 10.1093/jxb/erx116

**Published:** 2017-04-17

**Authors:** Jaime Puértolas, Elisabeth K. Larsen, William J. Davies, Ian C. Dodd

**Affiliations:** 1 The Lancaster Environment Centre, Lancaster University, Lancaster LA1 4YQ, UK

**Keywords:** ABA, drought, frequent irrigation, genotype screening, *Helianthus annuus*, phenotyping platform, *Populus nigra*, soil moisture heterogeneity.

## Abstract

Pot-based phenotyping of drought response sometimes maintains suboptimal soil water content by applying high-frequency deficit irrigation (HFDI). We examined the effect of this treatment on water and abscisic acid (ABA) relations of two species (*Helianthus annuus* and *Populus nigra*). Suboptimal soil water content was maintained by frequent irrigation, and compared with the effects of withholding water and with adequate irrigation. At the same average whole-pot soil moisture, frequent irrigation resulted in larger soil water content gradients, lower root and xylem ABA concentrations ([X-ABA]), along with higher transpiration rates or stomatal conductance, compared with plants from which water was withheld. [X-ABA] was not uniquely related to transpiration rate or stomatal conductance, as frequently irrigated plants showed partial stomatal closure compared with well-watered controls, without differing in [X-ABA] and, in *H. annuus*, [ABA]_leaf_. In two *P. nigra* genotypes differing in leaf area, the ratio between leaf area and root weight in the upper soil layer influenced the soil water content of this layer. Maintaining suboptimal soil water content alters water relations, which might become dependent on root distribution and leaf area, which influences soil water content gradients. Thus genotypic variation in ‘drought tolerance’ derived from phenotyping platforms must be carefully interpreted.

## Highlight

High-frequency deficit irrigation to maintain sustained low soil moisture results in wet upper soil layers which increase bulk root water potential and diminish ABA levels.

## Introduction

The study of plant responses to drought is becoming even more relevant under the current uncertainties regarding food and energy security under a changing climatic scenario. There is a growing pressure on regional water resources to maintain food and biofuel crop yield and an urgent need to increase agricultural production on marginal lands prone to drought conditions. Selecting drought-tolerant genotypes is one of the main research targets to increase crop yield under limited water availability (Chaves and Davies, 2010), but plant responses to water deficit are complex and the traits controlling those responses can have different impacts depending on the drought scenario (Tardieu, 2012). Thus assessing genotype performance under drought is problematic, especially when most studies simply withdraw water and quantify plant survival (Lawlor, 2013; Blum, 2014). Nowadays, powerful statistical tools can associate genes with specific phenological traits, including physiological responses to water deficit (Baginsky *et al.*, 2010; Hu and Xiong, 2014), which can improve the breeding process or even be used within genetic modification programmes. Technologically advanced high-throughput screening platforms equipped with automated irrigation systems can measure a wide range of morpho-physiological variables in a large number of genotypes to obtain the necessary data for those analyses (Furbank and Tester, 2011; Neilson *et al.*, 2015). However, the effects of experimental manipulations in controlled conditions on plant physiology, in particular those performed in potted plants, need to be carefully examined to determine whether similar physiological responses occur in field-grown plants (Poorter *et al.*, 2012, 2016). There are different approaches to restrict water when screening for drought tolerance (Passioura, 2012). The use of automated systems favours the use of frequent or continuous weighing of pots, with frequent irrigation aiming to restore a pre-determined pot weight corresponding to a specific soil moisture target, over sustained periods of time (Granier *et al.*, 2006; Pereyra-Irujo *et al.*, 2012; Tisne *et al.*, 2013). This method was discussed and defined by Blum (2011) as being of questionable physiological relevance in phenotyping genotypic variation in drought resistance.

Irrigation frequency can determine plant water relations and soil water availability. Under optimal conditions, high-frequency irrigation enhanced bulk soil water content, and therefore root water uptake efficiency and yield of lysimeter-grown sunflower (*Helianthus annuus*) plants (Segal *et al.*, 2006). Moreover, irrigation frequency at suboptimal soil moisture can greatly influence water relations of potted plants. Daily watering of potted *Pelargonium hortorum* plants with 50% of the water applied to well-watered control plants decreased abscisic acid (ABA) concentration in the xylem sap and increased transpiration rates and leaf water potential compared with plants receiving the same irrigation volume but applied cumulatively every 4 d (Boyle *et al.*, 2016*a*). Understanding the effects of different irrigation regimes within phenotypic screening is needed, as the method of imposing soil water deficit might modify genotype rankings of drought tolerance traits. In these experiments, since the volume of water added in each irrigation is proportional to the water lost since the previous irrigation, differences in water uptake between genotypes can determine soil moisture distribution and thus genotypic differences in drought responses. For example, genotypes with a larger leaf area, usually with higher water uptake, would need to receive more water in each irrigation event, which could change water distribution in the pot compared with genotypes receiving less water.

One of the physiological traits that may discriminate species differences in water use strategies is ABA production in response to soil drying (Sreenivasulu *et al.*, 2012). However, the effects of ABA on different aspects of plant physiology are complex and often contradictory (Saradadevi *et al.*, 2014), and finding an ABA ideotype (Blum, 2015) requires an understanding of how this hormone is produced and transported within the plant. Experiments inducing pronounced soil moisture heterogeneity by manipulating either irrigation placement (partial rootzone; Dodd *et al.*, 2008*a*) or timing (irrigation frequency experiments; Boyle *et al.*, 2016*b*) can diminish xylem ABA concentration, although other reports show that ABA levels might be regulated by overall soil water content (Einhorn *et al.*, 2012; Puértolas *et al.*, 2013). The impact of the irrigation procedure in phenotyping platforms needs to be assessed to understand the physiological impacts of different ABA levels.

This study aimed to determine the effect of frequent irrigation applied to maintain constant suboptimal soil water content in drought experiments on plant water and ABA relations. The objective was to assess the impact of this procedure on phenotype screening for drought resistance traits. For that purpose, two experiments in two different species compared plant physiological responses to three irrigation procedures: daily replacement of transpirational losses to ensure well-watered plants; periodic drying and complete rewetting cycles; and daily irrigation to maintain suboptimal soil moisture. Plants in the two deficit treatments were measured at the same whole-pot soil water content. Two contrasting species were chosen to assess the consistency of the observed responses. *Helianthus annuus* is a herbaceous species propagated from seeds described in previous reports as typically anisohydric, while *Populus nigra* is a woody isohydric species propagated from cuttings (Tardieu and Simonneau, 1998). The first experiment explored the effect of these treatments on *H. annuus* at different times of the day, and the second assessed these effects on two different genotypes of *P. nigra* with contrasting leaf area to test whether genotypic differences in plant water uptake influence the observed responses. Two questions were addressed. (i) For equivalent suboptimal soil moisture, how does high-frequency deficit irrigation (HFDI) modify water and ABA relations compared with plants subjected to drying cycles? (ii) Do genotypic differences in water use explain plant responses to HFDI?

## Materials and methods

Two experiments were performed. Experiment 1 assessed the effect of maintaining suboptimal soil moisture by applying HFDI on daily variation in ABA levels in *H. annuus.* Experiment 2 studied the effect of maintaining suboptimal soil moisture on ABA signalling in two genotypes of *P. nigra* with contrasting leaf area.

### Plant material, growing conditions, and irrigation treatments

In Experiment 1, two sunflower (*H. annuus*) seeds were sown in each of 64 square section pots (6×6×30 cm high; 1.1 litre volume), with a perforated (9 mm diameter holes) plastic sheet glued to one end to hold soil while allowing drainage. After germination, a single plant was left in each pot.

In Experiment 2, *P. nigra* hardwood cuttings from each of two genotypes were planted in wet perlite after dipping the basal end in a 1 mM indole-3-butyric acid solution to facilitate rooting. Genotypes were selected based on contrasting leaf morphology and total leaf area. Genotype B had larger leaves and higher total leaf area than genotype S. After 1 month, 32 rooted cuttings from each genotype were transplanted to cylindrical pots (6.5 cm in diameter, 21 cm in height, 0.8 litre volume) with a stainless steel mesh (0.7 mm aperture) at one end to assist drainage. The pot was designed to fit in the pressure chamber of the same volume.

In both experiments, pots were cut lengthwise in two halves and stuck together with duct tape to facilitate root and soil extraction at harvest. Pots were filled with an organic loam (John Innes No2, J. Arthur Bowers, UK) up to 3 cm from the top of the pot.

Plants were grown in a walk-in controlled environment chamber, under the following environmental conditions: photosynthetically active radiation (PAR)=400 µmol m^–2^ s^–1^ provided by halogen lamps (HQI-BT 400W/D, Osram, Germany); day/night temperature=24/16 °C; and photoperiod=16 h in Experiment 1, and 14 h in Experiment 2 to prevent excessive daily transpiration. At the beginning of the experiment, pots were watered to field capacity at the end of the photoperiod, left to drain overnight, and weighed to obtain weight at pot capacity (PW_sat_). Pots were weighed twice a day during the whole experiment and watered according to pot weight. Plant weight was neglected as it was <5% of the water weight at the lower soil moisture threshold.

**Fig. 1.**
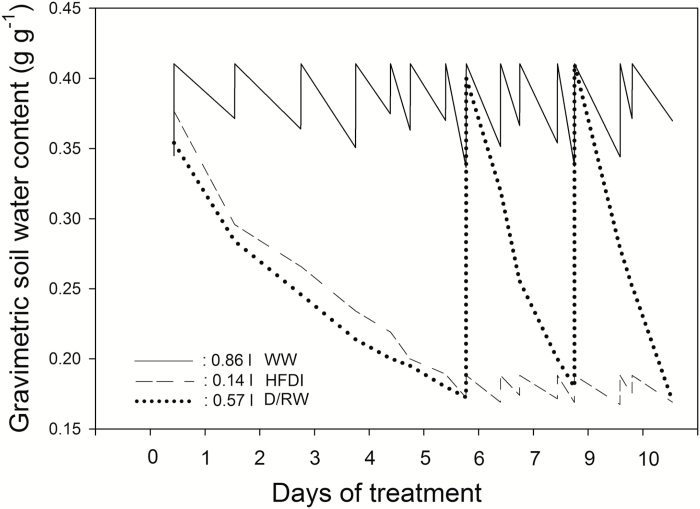
Evolution of whole-pot soil gravimetric water content (θ_g_) during Experiment 1 in one example plant of *Helianthus annuus* per irrigation treatment measured for 10 d after the start of the treatments (WW, continuous line; HFDI, dashed line; D/RW, dotted line). Total water applied for each treatment is shown. Measurements were taken at the end of the treatment application in all plants.

Plants were watered to PW_sat_ daily for the first 3 weeks in Experiment 1, and 2 weeks in Experiment 2. After that period, they were randomly assigned to the following three irrigation treatments (Fig.1).

#### WW

Plants were watered daily during the first 3 d and then twice a day to PW_sat_ (16 plants in Experiment 1; 12 plants in Experiment 2, six per genotype). Watering was carried out in the morning and after midday (2–3 h and 8–9 h after the start of the photoperiod, respectively).

#### D/RW

For the drying and rewetting cycle (24 plants in Experiment 1; 28 in Experiment 2, 14 per genotype), water was withheld until pot weight reached a threshold (a soil moisture that halved stomatal conductance (*g*_s_) compared with WW plants) and then re-watered to reach PW_sat._. In Experiment 1, this threshold was set at PW_sat_–275 g, which corresponded to an average soil water content of 0.16 g g^–1^ and a soil matric potential of –0.13 MPa according to a soil moisture release curve previously performed on the same substrate (Puértolas *et al.*, 2013). In Experiment 2, this threshold was PW_sat_–160 g (0.10 g g^–1^, corresponding to a soil matric potential of –1.09 MPa). Pots were weighed with the same frequency as WW plants, but watered only if weight was below the minimum threshold set. Plants were subjected to 2–3 cycles during the experiment.

#### HFDI

Water was withheld as in D/RW, and then plants were re-watered twice a day (as necessary) to reach the minimum threshold set in each experiment (24 plants in Experiment 1; 28 in Experiment 2, 14 per genotype).

### Measurement design

In Experiment 1, measurements were made over 4 d, starting 4 weeks after germination. On each day, two plants of D/RW and HFDI treatments (eight in total during the 4 d) and 1–2 plants of WW (5–6 plants in total) were measured immediately before the start of the photoperiod (pre-dawn), 1 h after the start of the photoperiod, immediately before the morning irrigation, and 7 h after the start of the photoperiod, immediately before the midday irrigation. WW and HFDI plants were randomly selected, while D/RW plants were selected according to pot weight to ensure they were below the PW_sat_–275 g threshold at the time of measurement, in order that whole-pot soil moisture was comparable with HFDI plants.

In Experiment 2, plants were measured over 6 d, starting 4 weeks after transplanting. For each measurement day, one plant per genotype of WW and 2–3 plants of D/RW and HFDI treatments were selected for measurements between 2 h and 7 h after the start of the photoperiod.

### Plant water use

In Experiment 1, *g*_s_ was measured in the two most apical fully expanded leaves with a porometer (AP4, Delta-T, Burwell, UK) and averaged. Measurements were not taken at pre-dawn.

In Experiment 2, for each plant, both ends of the pot were covered with duct tape, and pot weight was recorded 1 h before measurement. It was weighed again 1 h later to calculate the plant water uptake rate. Total leaf area was measured in both experiments with a leaf area meter (Li-3100C, Licor, Lincoln, NE, USA) and, in Experiment 2, plant transpiration rate was calculated as water uptake rate divided by leaf area. Preliminary *g*_s_ measurements were made at the beginning of the experiment. Since we found high variability across the canopy of each single plant, especially in the S genotype (data not shown), we decided not to measure it during the experiment and report only the whole-plant transpiration rate as a measure of stomatal control of water losses.

### Plant and soil water relations

One leaf from the upper third of the canopy (in Experiment 1, one of those where *g*_s_ was measured for morning and midday measurements) was excised, immediately frozen in liquid nitrogen, and stored at –20 °C. Then the plant was de-topped, and the shoot placed in a pressure chamber (Soil Moisture Equipment Corp., Santa Barbara, CA, USA) to measure shoot water potential (ψ_shoot_). In Experiment 2, pre-dawn leaf water potential was also measured in all harvested plants immediately before the start of the photoperiod (ψ_pd_) and pot weight was recorded simultaneously to estimate soil water content. After reaching the balancing pressure, an overpressure of up to 0.5 MPa was applied to allow sap collection. Sap was immediately frozen in liquid nitrogen and stored at –20 °C for subsequent determination of the ABA concentration in the shoot xylem sap ([X-ABA]_shoot_). Each pot was opened and roots were extracted from each of the three soil column layers (10 cm and 7 cm in length for Experiments 1 and 2, respectively). Roots were quickly washed (<60 s), blotted, frozen in liquid nitrogen, and stored at –20 °C. A soil sample of each layer was weighed, oven-dried at 70 °C until constant weight was reached, and weighed again to calculate soil gravimetric water content in each section (θ_g_).

Additionally, in Experiment 2, after shoot removal to measure ψ_shoot_, half of the pots (three WW and seven D/RW or HFDI for each genotype) were inserted in the pressure chamber to determine bulk root water potential (ψ_bulkroot_) and extract root xylem sap. After measuring ψ_bulkroot_, pressure was increased at 0.04 MPa intervals. At each step, sap was collected for 20 s in a pre-weighed Eppendorf tube and weighed to calculate the sap flow rate. This sap flow rate was compared with the actual whole-plant transpiration rate and pressure was increased until the two values matched. Sufficient sap (>50 µl) was collected at the matching flow rate to determine root xylem ABA concentration ([X-ABA]_root_). The remaining pots, which were not pressurized, were opened and a root sample (2 cm in length) was excised from each of three layers within the soil column (7 cm in length), tapped to remove adhering soil particles, blotted, and quickly inserted (<15 s between excision and insertion) in a psychrometric chamber (C-52, Wescor, Logan, UT, USA) to equilibrate before measuring ψ_root_. Measurements were made by dew-point psychrometry. Each psychrometric chamber was previously calibrated by determining the voltage output versus water potential relationship using four different NaCl concentrations of known osmotic potential.

### ABA determination

Stored leaf and root tissues were freeze-dried and finely ground to determine [ABA]_leaf_ and [ABA]_root_. Root dry weight in each layer was determined before grinding. The ground tissue was incubated in distilled water (1:50, w/w) at 4 °C overnight in a shaker. ABA concentrations in xylem sap and aqueous extracts of tissues were analysed by a radioimmunoassay (Quarrie *et al.*, 1988). Based on a cross-reactivity test (Quarrie *et al.*, 1988), there was no non-specific interference during the assay for either leaf or root extracts of *P. nigra* and *H. annuus*.

### Statistical analyses

For both experiments, variables not associated with position in the soil column were analysed by two-way ANOVA, with the factors comprising irrigation treatment (both experiments), time of the day for Experiment 1 and genotype for Experiment 2, with day of measurement as a block. Variables associated with vertical position in the soil column ([ABA]_root_, root dry weight, θ_g_, ψ_root_) were analysed by repeated measures ANOVA, with soil layer as the repetition factor. Additionally, the possible effect and interactions of pressurization on [ABA]_root_ in Experiment 2 were analysed by introducing that factor in the analysis. Since neither pressurization nor the interactions with treatment or soil layer were significant, data for both pressurized and non-pressurized pots were included in the analysis. When interactions were significant, separate one-way ANOVAs were applied comparing irrigation treatments in each of the levels of the other factor. To account for variability in plant leaf area in Experiment 2, one-way analysis of covariance (ANCOVA) was performed, with leaf area as covariate and irrigation as a factor using only the two water deficit treatments. One-way ANCOVA was also used to compare the slope of the relationship of ψ_root_ and [X-ABA]_root_ with transpiration rate between two data sets: WW+HFDI and WW+D/RW. In all cases, a post-hoc test discriminated differences between irrigation treatments (Tukey HSD, *P*<0.05).

## Results

### *Experiment 1.* Helianthus annuus

At harvest, leaf area was higher (*P*<0.001) in WW (572 ± 32 cm^2^) than in D/RW (353 ± 26 cm^2^) and HFDI (343 ± 26 cm^2^) plants, with no significant differences between the two latter treatments.

**Fig. 2.**
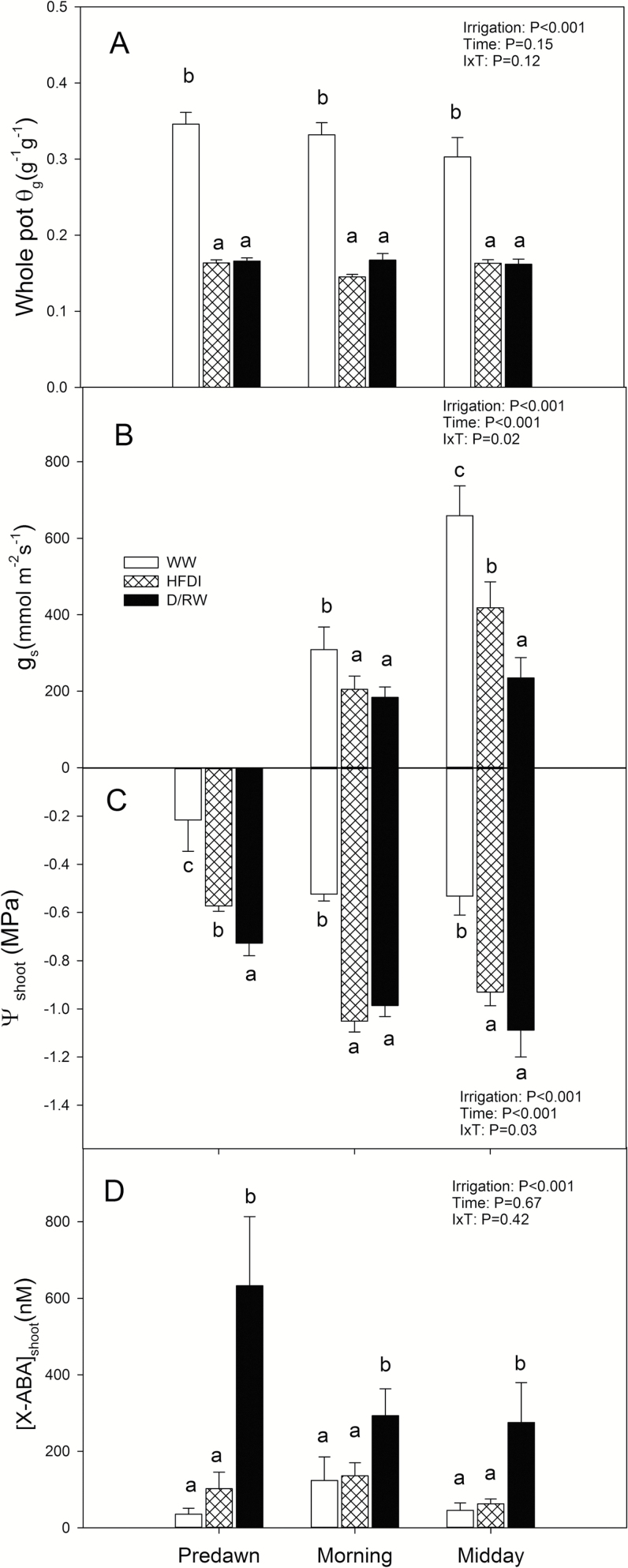
Whole-pot gravimetric soil water content (θ_g_)(A), stomatal conductance (*g*_s_) (B), shoot water potential (ψ_shoot_) (C), and ABA concentration in shoot xylem sap ([X-ABA]_shoot_) (D) in *Helianthus annuus* in different irrigation treatments (HFDI, patterned; D/RW, black; WW, white) at different times of the day. Data are means ±SE of eight replicates for HFDI and D/RW, and six for WW. *P*-values from the ANOVA are shown for each variable. Different letters denote significant differences between irrigation treatments within each time of the day (Tukey, *P*<0.05)

Whole-pot soil water content was 50% lower in D/RW and HFDI compared with WW, but values were similar in D/RW and HFDI at any time of the day, as intended (Fig. 2A). *g*_s_ was higher at midday than in the morning (Fig. 2B). WW plants had higher *g*_s_ than the other treatments, but differences were larger at midday. Morning measurements revealed no statistical differences in *g*_s_ between HFDI and D/RW, but at midday HFDI had an 80% higher *g*_s_ than D/RW. Irrigation, time of the day, and their interaction significantly affected ψ_shoot_, which was always higher for WW plants independent of the time of measurement (Fig. 2C). Pre-dawn ψ_shoot_ was 0.15 MPa lower for D/RW than HFDI plants, but there were no significant differences in ψ_shoot_ in the morning and at midday. Foliar ABA concentration was significantly higher (*P*=0.001) in D/RW plants (6.2 ± 0.9 nmol g^–1^ DW) than in WW and HFDI plants, which showed similar values (3.0 ± 0.5 nmol g^–1^ DW and 2.9 ± 0.4 nmol g^–1^ DW for WW and HFDI, respectively). Leaf xylem ABA concentrations showed similar patterns to foliar ABA accumulation (Fig. 2D). Although treatment effects on *g*_s_ and ψ_shoot_ varied according to the time of day (Fig. 2B, C), xylem ABA concentration was always higher in D/RW than in WW and HFDI plants.

**Fig. 3.**
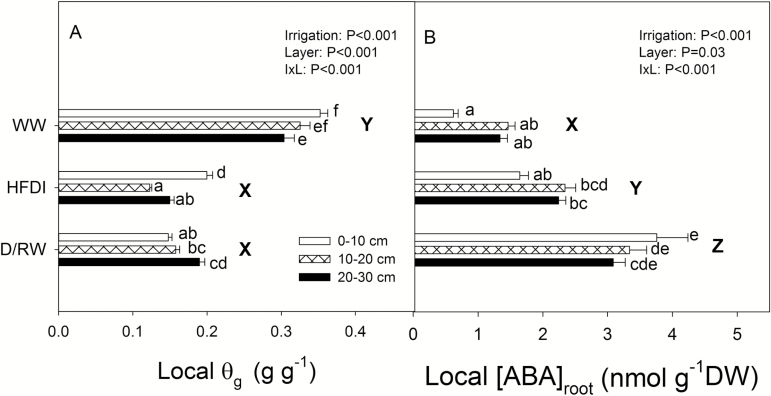
Soil gravimetric water content (θ_g_) (A) and ABA concentration in roots ([ABA]_root_) (B) in *Helianthus annuus* in different irrigation treatments and soil layers (0–10 cm, white bars; 10–20 cm, patterned bars; 20–30 cm, black bars). Data are means ±SE of eight replicates for HFDI and D/RW, and six for WW. *P*-values for irrigation, soil layer, and their interaction in the repeated measures ANOVA are shown for each variable. Time and its interactions were not significant for either variable. Different lower case letters denote significant differences between depth×irrigation treatment combinations, while upper case letters denote differences of the average across depths between irrigation treatments (Tukey, *P* < 0.05).

**Fig. 4.**
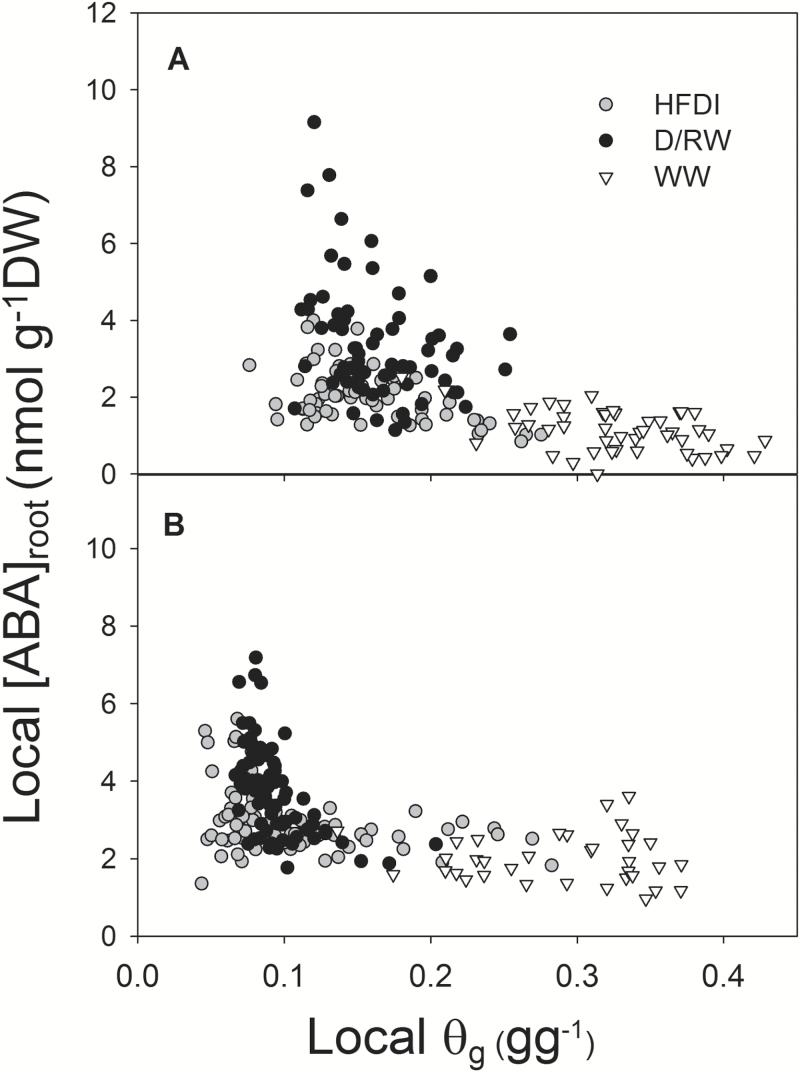
Relationship between local soil gravimetric water content (θ_g_) and ABA concentration in the roots ([ABA]_root_) for each irrigation treatment (HFDI, grey circles; D/RW, black circles; WW, white triangles) in *Helianthus annuus* (A) and *Populus nigra* (B). Each point represents paired samples taken within a layer within a plant.

Soil moisture varied between soil layers in all treatments, with the highest soil water content in the upper 10 cm for WW and HFDI plants, while in D/RW plants the basal 10 cm had a slightly higher θ_g_. Root dry weight was higher in the upper 10 cm (Table 1). Averaging across soil layers, [ABA]_root_ decreased in the order: D/RW>HFDI>WW (Fig. 3B). However, a strong irrigation×layer interaction was observed. In WW and HFDI, [ABA]_root_ was lower in the upper 10 cm than in the other two lower layers, while in D/RW there were no significant differences across soil layers. For a similar level of local soil water content, [ABA]_root_ in HFDI was lower than in D/RW (Fig. 4A). No statistical differences between time of the day were found for θ_g_, root dry weight, or [ABA]_root._

**Table 1.** Root dry weight (mean ±SE; mg) in each column layer for the three irrigation treatments and two species In parentheses, the percentage of the root dry weight in the layer with respect to total dry weight is given.

		Upper layer	Middle layer	Lower layer	Total
*H. annuus*	WW	40.5 ± 4.9 a	30.8 ± 4.1 a	33.7 ± 4.0 a	105.0 ± 12.0 A
		(40.0 ± 2.3 b)	(28.6 ± 1.8 a)	(31.4 ± 2.1 a)	
	HFDI	29.9 ± 4.2 b	21.8 ± 3.5 a,b	18.0 ± 3.4 a	69.7 ± 7.5 A
		(42.4 ± 2.9 b)	(32.1 ± 2.1 a)	(25.5 ± 2.1 b)	
	D/RW	32.0 ± 4.2 a	27.6 ± 3.5 a	26.1 ± 3.4 a	85.7 ± 10.3 A
		(39.2 ± 2.6 b)	(31.0 ± 1.6 a)	(29.7 ± 2.5 a)	
	Average	33.5 ± 2.6 b	26.3 ± 2.1 a,b	25.1 ± 2.2 a	
		(40.6 ± 1.3 b)	(30.8 ± 1.1 a)	(28.6 ± 1.5 a)	
*P. nigra*	WW	239 ± 30 a,b	178 ± 18 a	334 ± 46 b	750 ± 70 A
		(31.8 ± 2.7 a)	(23.9 ± 1.5 a)	(44.4 ± 3.7 b)	
	HFDI	264 ± 35 b	158 ± 12 a	338 ± 22 c	760 ± 39 A
		(35.7 ± 2.2 b)	(20.4 ± 0.9 a)	(43.8 ± 2.1 c)	
	D/RW	294 ± 28 b	206 ± 18 a	406 ± 28 c	904 ± 60 B
		(31.9 ± 1.7 b)	(22.4 ± 1.2 a)	(45.7 ± 1.4 c)	
	Average	271 ± 15 b	181 ± 10 a	365 ± 17 c	
		(33.4 ± 1.3)	(21.9 ± 0.7 a)	(45.7 ± 1.2 c)	

For each species, different lower case letters denote statistical differences between layers within each irrigation treatment and within the average across treatments. Upper case letters denote differences between irrigation treatments for total weight (Tukey, *P*<0.05)

### *Experiment 2.* Populus nigra

Leaf area was 36% higher in genotype B than in genotype S (Table 2) and tended to be smaller (12%) in HFDI than in D/RW and WW plants.

**Table 2.** Results of the ANOVA for variables measured in Experiment 2 which are not shown in the figures

Variable	Source of variation	df	*F*	*P*
Leaf area	Irrigation	2	3.0	0.06
	Genotype	1	32.9	**<0.001**
	I×G	2	0.9	0.40
ψ_predawn_	Irrigation	2	10.7	**<0.001**
	Genotype	1	5.1	**0.03**
	I×G	2	1.7	0.14
[ABA]_leaf_	Irrigation	2	3.6	**0.03**
	Genotype	1	0.5	0.49
	I×G	2	0.8	0.46

ψ_predawn_, pre-dawn water potential; [ABA]_leaf_, ABA concentration in leaf tissue.

In bold, statistical significance (*P*<0.05).

ψ_pd_ was higher in WW (–0.14 ± 0.03 MPa) than in HFDI (–0.42 ± 0.07 MPa) and lowest in D/RW (–0.67 ± 0.08 MPa) (Table 2), even though whole-pot soil water content at the time of measurement was higher in D/RW than in HFDI (but HFDI had higher local soil water content in the upper layer). Averaged across treatments, ψ_pd_ was 0.25 MPa lower in genotype B than in genotype S, even though whole-pot soil water content was similar (*P*=0.67) between genotypes.

**Fig. 5.**
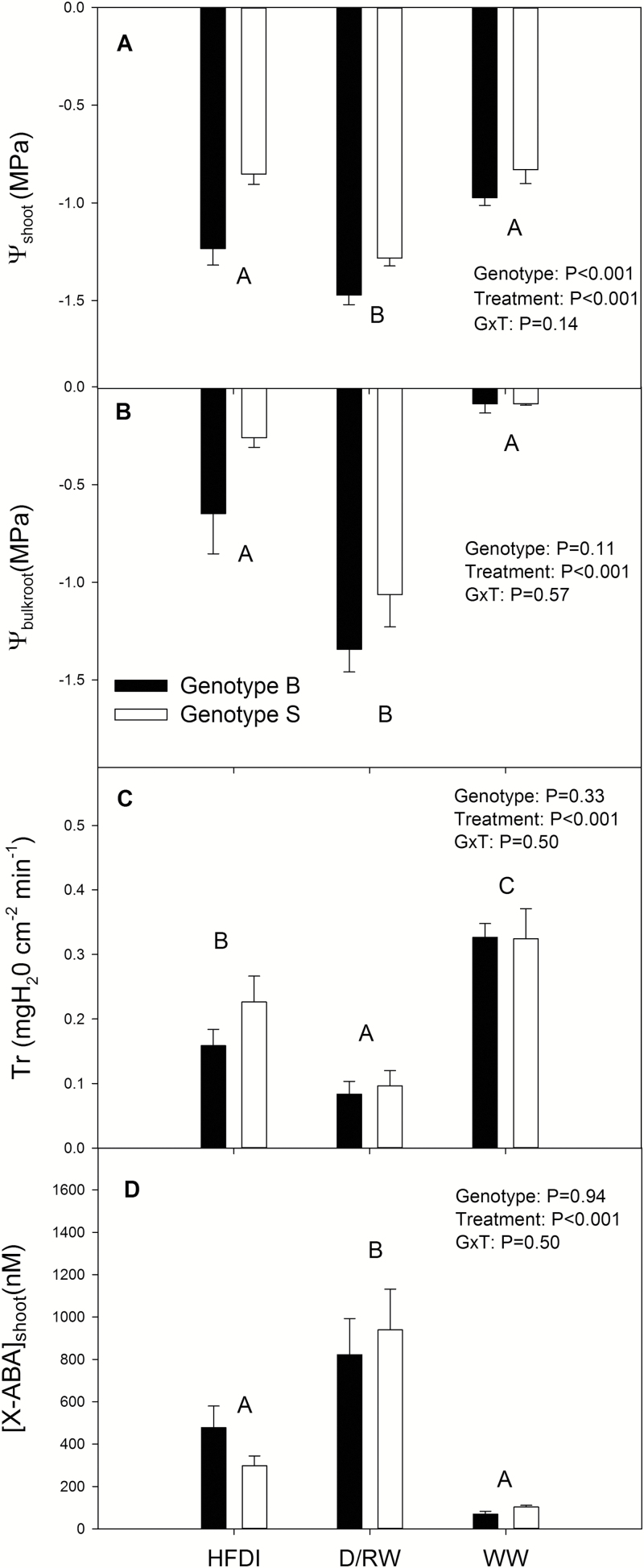
Shoot water potential (ψ_shoot_) (A), bulk root water potential (ψ_bulkroot_) (B), transpiration rate (T_r_) (C), and ABA concentration in shoot xylem sap ([X-ABA]_shoot_) (D) in *Populus nigra* in different irrigation treatments and genotypes (genotype B, black bars; genotype S, white bars). Data are means ±SE of 14 replicates for HFDI and D/RW, and 6 for WW, except for ψ_root_, where *n*=7 for HFDI and D/RW, and *n*=3 for WW. *P*-values from the ANOVA are shown for each variable. Different letters denote significant differences between irrigation treatments for the average of the two genotypes, as the genotype×irrigation interaction was not significant for any variable (Tukey, *P*<0.05).

Overall variability in both ψ_pd_ and ψ_bulkroot_ within HFDI *P. nigra* plants was much higher than in ψ_pd_ for *H. annuus*, with a higher coefficient of variation (CV) for *P. nigra* (CV=0.93 for both ψ_pd_ and ψ_bulkroot_ in *P. nigra*; CV=0.11 for ψ_pd_ in *H. annuus*). Genotype did not alter the responses of shoot and bulk root water potential to the irrigation treatments (no significant genotype×treatment interaction). Although genotype did not affect ψ_bulkroot_, ψ_shoot_ was –0.25 MPa lower in genotype B (averaged across treatments). Both ψ_shoot_ and ψ_bulkroot_ were lowest in the D/RW treatment, with no significant differences between HFDI and WW plants (Fig. 5A, B).

**Fig. 6.**
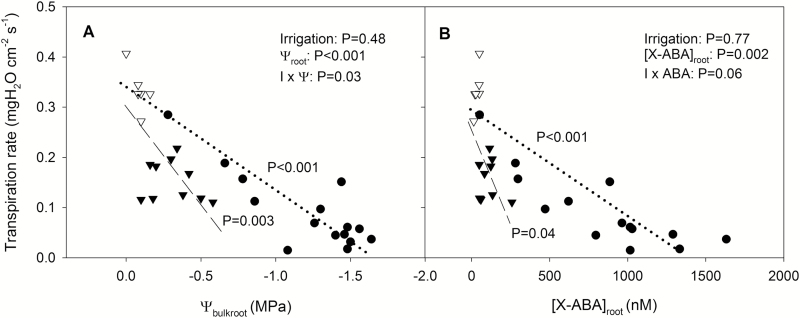
Relationship between transpiration rate and (A) bulk root water potential (ψ_bulkroot_) and (B) ABA concentration in the shoot xylem sap ([X-ABA]_shoot_) for each irrigation treatment (HFDI, black triangles; D/RW, black circles; WW, white triangles) in *Populus nigra*. The regression line for WW and HFDI (dashed) and WW and D/RW (dotted) pools is shown in both panels. *P*-values and the results of the ANCOVA to compare regression slopes are shown.

Transpiration rate (normalized per unit leaf area) was affected by irrigation treatment, but not by genotype or genotype×treatment interaction. Transpiration of HFDI and D/RW plants was 30% and 60% of WW plants, respectively (Fig. 5C). Foliar ABA concentration ([ABA]_leaf_) was lower (*P*=0.03) in WW (6.2 ± 0.4 nmol g^–1^ DW) than in D/RW (8.3 ± 0.6 nmol g^–1^ DW) and HFDI (8.1 ± 0.4 nmol g^–1^ DW) plants. ABA concentration in shoot xylem sap ([X-ABA]_shoot_) was higher only in D/RW, with no statistical differences between HFDI and WW (Fig. 5D), even though average [X-ABA]_shoot_ was 2- to 3-fold higher in HFDI, due to great variability within this treatment. Transpiration rate decreased with both ψ_bulkroot_ and [X-ABA]_root_. However, the slope of the decrease with respect to WW was steeper in HFDI than in D/RW (Fig. 6).

**Fig. 7.**
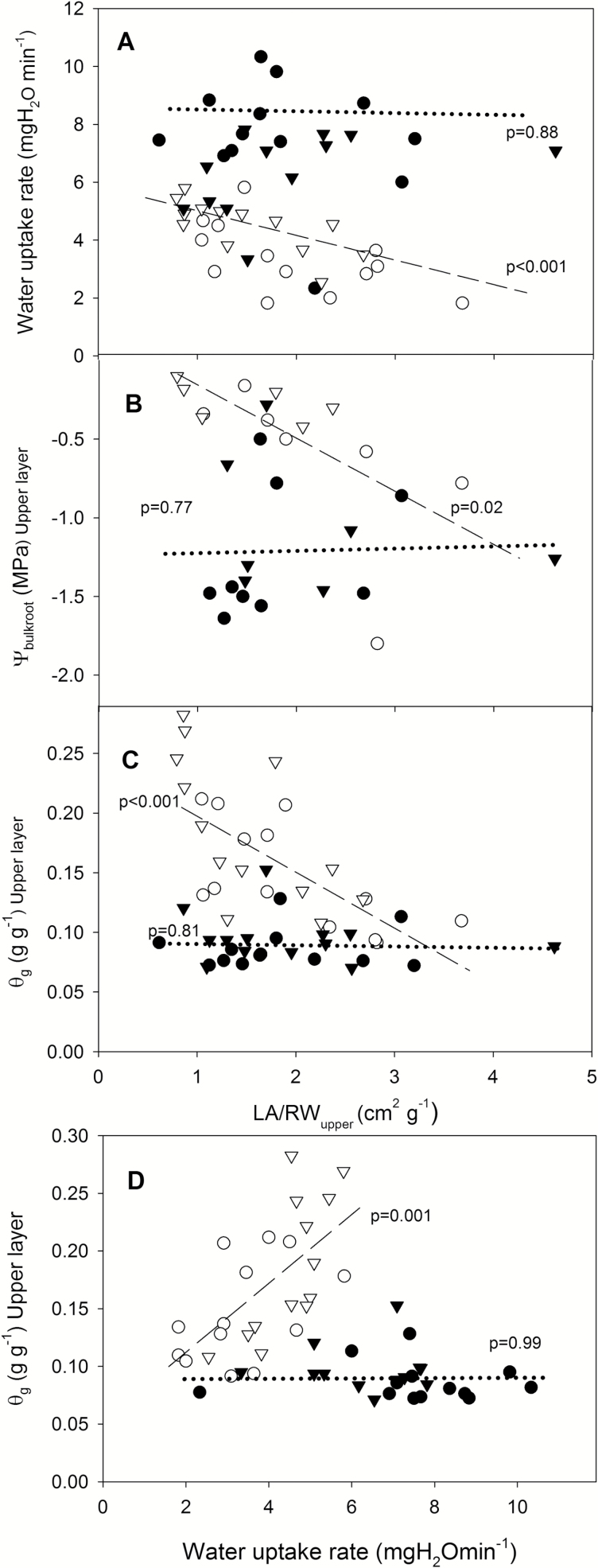
Relationships between the leaf area to root dry weight ratio in the 0–7 cm upper soil layer (LA/RW_upper_) and total plant water uptake (A), bulk root water potential (ψ_bulkroot_) (B), and soil gravimetric water content (θ_g_) in that layer (C), and between plant water uptake and θ_g_ in the upper layer (D) for each of the two deficit irrigation treatment (HFDI, white symbols; D/RW, black symbols) and genotypes (B, circles; S, triangles) at the end of the experiment in *Populus nigra*. For both irrigation treatments, the fitted linear regression line and the *P*-values of the regression are shown (*P*<0.05 in the ANCOVA; HFDI, dashed; D/RW, dotted).

Leaf area did not affect transpiration rate. However, within the two deficit irrigation treatments, water uptake was related to the leaf area:root weight ratio, but the relationship differed (*P*=0.01 for the leaf area×treatment interaction). While water uptake was not correlated with the leaf area:root weight ratio in D/RW, it decreased in HFDI (Fig. 7A). Plant water uptake was also positively related to soil moisture in the upper soil layer (0–7 cm) for HFDI but not for D/RW (*P*=0.001 for the water uptake×treatment interaction) (Fig. 7D). Consequently, ψ_bulkroot_ (Fig. 7B) and soil moisture in the upper layer (Fig. 7C) decreased with the leaf area:root weight ratio in HFDI, while no relationship was observed in D/RW. For the two lower layers, soil moisture was not related to either leaf area or water uptake. Overall, these results suggest that the leaf area:upper root weight ratio determined soil moisture gradient, ψ_bulkroot_, and, in turn, transpiration rate.

**Fig. 8.**
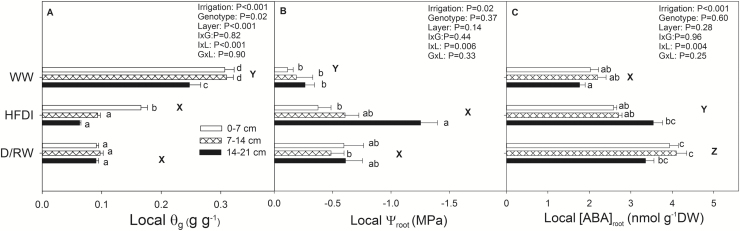
Soil gravimetric water content (θ_g_) (A), root water potential (ψ_root_) (B), and ABA concentration in roots ([ABA]_root_) (C) in *Populus nigra* in different irrigation treatments and soil layers (0–7 cm, white bars; 7–14 cm, patterned bars; 14–21 cm, black bars). Data are means ±SE of 14 replicates for HFDI and D/RW, and 6 for WW, except for ψ_root_, where *n*=7 for HFDI and D/RW, and *n*=3 for WW. *P*-values for irrigation, genotype, layer, and their two-way interactions in the repeated measures ANOVA are shown for each variable (no triple interaction was statistically significant). Different lower case letters denote significant differences between depth×irrigation treatment combinations, while upper case letters denote differences of the average across depths between irrigation treatments (Tukey, *P*<0.05).

Whole-pot soil water content was higher in WW and similar in D/RW and HFDI (*P*<0.001). It was significantly higher in genotype S (*P*=0.02), with no genotype×irrigation interaction (*P*=0.83). Vertical soil moisture profiles differed between treatments (a highly significant soil layer×irrigation treatment interaction for θ_g_; *P*<0.001). Soil moisture was similar in all layers for D/RW plants, but strongly decreased from the upper wetter layer to the drier lower layer in HFDI plants (Fig. 8A). Root dry weight distribution was similar across irrigation treatments: lowest in the middle and highest in the lower layer (Table 1). Genotype B had 46% more root biomass than genotype S (*P*<0.001), with a greater difference in the lower layer (*P*=0.001 for depth×genotype interaction). D/RW plants had 20% more root biomass than the other two treatments.

Root water potential did not differ between soil layers in WW and D/RW plants, and was on average 0.4 MPa higher in WW plants. There was a pronounced ψ_root_ gradient within the soil column in the HFDI treatment (Fig. 8B), with the upper layer having a similar ψ_root_ to WW plants. Root ABA concentration was highest in D/RW and lowest in WW plants (Fig. 8C), with no genotype or genotype×irrigation interaction. In HFDI, it was 30% higher in the lower layer than in the other two layers, which had similar values to WW plants. In D/RW and WW plants, [ABA]_root_ was lower in the lower layer of the column. In contrast to sunflower, [ABA]_root_ increased in the driest layers similarly in HFDI and D/RW.

## Discussion

**Fig. 9.**
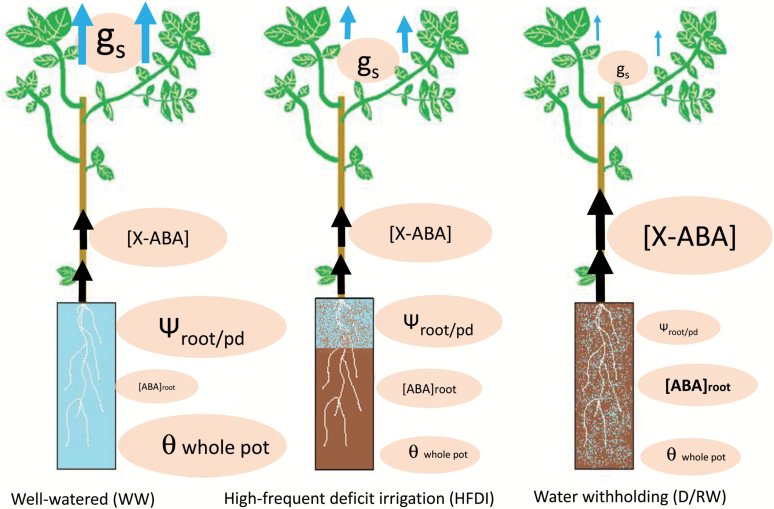
Graphic representation of the common main physiological effects of high-frequency deficit irrigation (HFDI) compared with optimal irrigation (WW) and withholding water (D/RW) observed in this experiment. The relative effect is represented by the size of the ovals and, for *g*_s_ and [X-ABA], arrows. The effect on moisture in different soil layers is represented by different grey tones and textures (light grey>textured light grey>textured dark grey>dark grey). [X-ABA], ABA concentration in xylem sap; [ABA]_root_, root ABA concentration averaged across the whole root system; ψ_root/pd_, root (or pre-dawn) water potential; *g*_s_, stomatal conductance; θ_whole pot_, whole-pot soil water content. (This figure is available in colour at *JXB* online.)

HFDI increased ψ_root_ and decreased [X-ABA]_shoot_ in comparison with plants from which water was withheld, at the same whole-pot soil moisture content (Fig. 9). Thus this irrigation procedure might not be appropriate when screening for stomatal responses to drought, as it improves root water status and suppresses xylem ABA concentration. Therefore, the results from screening using HFDI must be taken with caution. For example, the low [X-ABA]_shoot_ in sunflower was not related to decreased *g*_s_ in comparison with well-watered plants (cf. Fig. 2B, D), in contrast to previous predictions for sunflower (Tardieu and Simonneau, 1998) where [X-ABA]_shoot_ is uniquely related to *g*_s_. Even though the radioimmunoassay method used to quantify ABA concentration might not as readily detect subtle differences unlike more recent methods based on HPLC (McAdam, 2015), the clear differences between drought treatments found in this study demonstrate this differential effect of HFDI on the ABA versus stomatal conductance relationship. Moreover, since ψ_bulkroot_ or ψ_pd_ determine *g*_s_ (Fig. 6A) and other physiological traits (such as [ABA]_root_), different effects of HFDI in different genotypes can impact on phenotype screening for drought tolerance. While root water status within the HFDI treatment was consistent in sunflower, individuals were highly variable in poplar as the higher CV for root and pre-dawn water potential indicates. The ratio between leaf area and root weight explained part of this variability in HFDI plants (Fig. 7B), which in turn determined water uptake (Fig. 7A), but this ratio had no impact in D/RW plants. Thus HFDI plants with a high leaf area to upper root ratio had similar soil moisture in the upper layer and root water status to D/RW, while low ratio plants were similar to WW plants. This suggests that using this irrigation procedure to screen for drought tolerance, when leaf area or root allocation differs between genotypes, simply reflects morphological differences that affect soil water content of the upper layers and therefore root water potential. Genotypes with lower leaf area and a higher proportion of roots in the upper soil layers maintained higher transpiration rates and shoot water potential under suboptimal soil moisture, while in many drought scenarios, deep rooting accounts for drought tolerance (Puértolas *et al.*, 2014; Lynch and Wojciechowski, 2015).

As expected, frequent irrigation was necessary to maintain a pre-determined suboptimal soil water content in the pot (Fig. 1), which altered the vertical distribution of soil moisture within the soil column in contrast to pots at the same overall water content (weight) from which water was withheld (Figs 3A, 8A). The small volume of water added in each event was presumably taken up by the upper part of the root system, preventing its drainage to basal soil layers. Moreover, as soil dries, its hydraulic conductivity drops sharply (Lobet *et al.*, 2014), further preventing water movement from upper to lower soil layers. Since deeper roots depleted soil water that was not replaced by irrigation, deeper soil layers became drier in the HFDI treatment (Figs 3A, 8A). This effect was more pronounced in *Populus*, where root weight was higher in the basal layer, than in sunflower, with more roots in the upper part (Table 1). Soil moisture distribution in HFDI plants differed from that observed in plants from which water was withheld. Species differences in soil water distribution within this treatment (D/RW) may be partly explained by root distribution. In sunflower (higher root weight in the upper part), soil moisture tended to be higher in the bottom in opposition to HFDI, while in poplar (higher root allocation at the bottom) soil water content was homogeneous (Table 1). Interactions between root and soil moisture distribution determine spatial patterns of water uptake (Lobet *et al.*, 2014), along with species- or genotype-specific traits such as hydraulic architecture (Draye *et al.*, 2010), in a feedback mechanism that influences soil moisture distribution. Together with the potential impact of genotypic differences in biomass allocation (Table 1) on the creation of soil moisture gradients detailed above, this could obscure the results of species or genotype screening for water use strategies when applying high-frequency deficit irrigation to maintain low soil water content in phenotyping platforms. As an example, when comparing the drought responses of two *Populus* genotypes using this irrigation approach, [X-ABA] increased coincident with lower ψ_pd_ and lower transpiration in the genotype with higher leaf area (Chen *et al.*, 1997). According to our results, that could not be interpreted as superior drought tolerance of this genotype but instead a consequence of faster water depletion.

Pronounced soil moisture gradients greatly affected root ABA accumulation, and this impact varied with species. In *H. annuus*, local ABA accumulation in response to soil drying was much lower when a wet layer was present (Fig. 4A), as in *Phaseolus vulgaris*, which was attributed to redistribution of water within the root (Puértolas *et al.*, 2013). However, in poplar, [ABA]_root_ responded to local soil drying more similarly in both drought treatments (Fig. 4B). Although water redistribution from upper to lower roots driven by root water potential gradients is well documented (Burgess *et al.*, 2001), the predominant pathways of this water movement are not well known. However, assuming similarity with the normal upward water flow, decreased radial conductivity in the endodermis might reduce the extent of the redistribution by limiting the reverse flow of water from the xylem vessel to the cortex. The higher ABA accumulation and lower root water potential in the lower layer of *P. nigra* might be explained by higher suberization of the endodermis of woody compared with herbaceous species (Steudle, 2000), which may have impeded water redistribution within the root. Regardless of ABA accumulation patterns, ABA export from roots in the dry low layers to the shoots might be low (Boyle, 2015), in agreement with the predictions of models explaining root to shoot ABA signalling in heterogeneous soil as a function of both ABA accumulation and the distribution of water uptake within the rootzone (Dodd *et al.*, 2008*b*; Puértolas *et al.*, 2016), as confirmed by the low [X-ABA] of HFDI plants (Figs 2D, 5D). As observed previously (Khalil and Grace, 1993; Puértolas *et al.*, 2013), root ABA accumulation averaged across the whole root system and xylem ABA concentration followed the same pattern across irrigation systems in both species (Table 3, Fig. 9).

**Table 3.** Effect of high-frequency deficit irrigation (HFDI) on different variables compared with the effect of optimal irrigation (WW) and withholding water (D/RW) in three species

**Variable**	Treatment	*Helianthus annuus*	*Populus nigra*	*Solanum lycopersicum*
[X-ABA]	WW	NS	NS	NS
	D/RW	+290%	+130%	+306%
[ABA]_root_	WW	–45%	–29%	NS
	D/RW	+63%	+28%	+249%
ψ_root/pd_	WW	+61%	+80%	^*a*^
	D/RW	–28%	–157%	–285%
*g*_s_/Tr	WW	+56%	+74%	+136%
	D/RW	–24%	–53%	–55%

Data from *H. annuus* and *P. nigra* are from the present study, while for *S. lycopersicum* the data were extracted from Boyle (2015), where watering with 100% (WW) and 50% of the potential evapotranspiration applied either daily (HFDI) or every 3 d (D/RW) were compared. [X-ABA], ABA concentration in shoot (*H. annuus*, *P. nigra*) or leaf (*S. lycopersicum*) xylem sap; [ABA]_root_, ABA concentration in root tissue averaged across the whole root; ψ_root/pd_, root (*P. nigra*, *S. lycopersicum*) or pre-dawn leaf (*H. annuus*) water potential; *g*_s_/Tr, stomatal conductance (*H. annuus*, *S. lycopersicum*) or transpiration rate (*P. nigra*).

Values shown represent the percentage change in each treatment compared with the HFDI according to the formula: Change=100×[(Treat–HFDI)/HFDI], where Treat is the mean value of the considered variable for that irrigation treatment and HFDI the mean value for the HFDI treatment. Therefore, positive values indicate that the treatment was greater than the HFDI treatment, while negative values indicate that the treatment was less than the HFDI treatment. NS, non-significant differences between the treatment and HFDI. ^*a*^It was not possible to calculate ψ_root_ for WW, since positive pressure was observed in all plants.

The light levels under which the experiments were conducted (400 µmol m^–2^ s^–1^) may have attenuated the effects of the two drought treatments on stomatal conductance, as the effect of water deficit on *g*_s_ is magnified at higher light intensities (Gimenez *et al.*, 1992; Yin *et al.*, 2006). Nevertheless, both treatments decreased *g*_s_ or plant transpiration rate, which could not be uniquely related to shoot water status or xylem or leaf ABA concentration in both species (Figs 2, 5, 6B). On the contrary, they followed the same pattern across irrigation treatments as ψ_bulkroot_ or ψ_pd_ in both species, with intermediate values of *g*_s_ or transpiration rate in HFDI plants compared with WW (higher) and D/RW (lower) explained by intermediate values of ψ_pd_ or ψ_bulkroot_ (Fig. 2B, C and 5B, C, respectively). However, treatment variation in the relationship between ψ_bulkroot_ and transpiration rate in poplar (Fig. 6A) suggests decoupling between root water status and stomatal aperture, probably due to higher leaf ABA levels in HFDI plants of this species. Nevertheless, the coincidence between root water status and water use suggests the existence of a root-sourced signal controlled by overall root water status and not directly related to [X-ABA], which regulates stomatal aperture. Several candidates have been identified including changes in xylem pH and other hormones or ions (Davies *et al.*, 2002; Ernst *et al.*, 2010). Recent reports also suggest that drought-induced suppression of strigolactone synthesis increases stomatal sensitivity to ABA (Visentin *et al.*, 2016). The nature of this chemical long-distance signal has attracted debate, and different mechanisms of its effect in the plant and the interaction with hydraulic signal have been proposed (Tardieu, 2016). The soil moisture gradients generated with HFDI in species where shoot water status is not altered by soil drying (such as tomato or poplar) might be an appropriate system to study stomatal regulation by non-ABA root-sourced signals and the interaction with hydraulic signals since the existence of a wet layer attenuates the ABA signal while the dry soil still decreases stomatal conductance. Moreover, this irrigation approach could be better suited to screen for tolerance to specific drought scenarios, where large soil moisture gradients are present and genotypic variability in non-ABA root-sourced chemical or hydraulic signals are important. For example, this approach could help discriminate the most drought tolerant within a selection of deep-rooted genotypes. Therefore, even though our results indicate certain limitations to the use of phenotyping platforms to screen for drought tolerance, they still can be very useful to understand the physiological effects of different irrigation procedures.

### Conclusions

When long-term experiments to assess crop drought tolerance maintain constant, suboptimal whole-pot soil water content via high irrigation frequency, large soil moisture gradients are created within the pot, with wet upper and very dry lower layers. This treatment consistently limited root ABA accumulation (Fig. 4) and suppressed long-distance ABA signalling (same [X-ABA] as WW plants but much lower than in D/RW plants; Fig. 9; Table 3). Thus *g*_s_ was better related to ψ_root_ than to [X-ABA], suggesting that another root-sourced chemical signal induced partial stomatal closure. Shoot water potential did not seem responsible, since ψ_shoot_ responses varied between species and were not related to *g*_s_ (Figs 2, 5). All these physiological responses depended on moisture content of the uppermost soil layer which in turn was influenced by the plant water uptake rate (Fig. 7), which might invalidate the use of the high-frequency deficit irrigation in genotype screening for drought tolerance.

## Abbreviations:

ABA: abscisic acid
[ABA]_leaf_: abscisic acid concentration in leaf tissue
[ABA]_root_: abscisic acid concentration in root tissue
D/RW: dry and rewetting irrigation treatment
*g*_s_: stomatal conductance
HFDI: high-frequency deficit irrigation
PW_sat_: pot weight at soil saturation
[X-ABA]_root_: abscisic acid concentration in root xylem sap
[X-ABA]_shoot_: abscisic acid concentration in shoot xylem sap
WW: well-watered irrigation treatment
ψ_bulkroot_: bulk root water potential
ψ_root_: local root water potential
ψ_pd_: pre-dawn leaf water potential
ψ_shoot_: shoot water potential.
